# The Visit of French Doctors to London

**Published:** 1905-01-21

**Authors:** 


### THE VISIT OF FRENCH DOCTORS TO LONDON.

*Concluded from page 272.*

VIII.—DERMATOLOGY IN THE HOSPITALS. By DR. GASTON.

This article is mainly descriptive and makes only occasional comments. Its contents, therefore, are rather of special than of general interest, dealing with the treatment adopted at the different hospitals in connection with diseases of the skin. Attention is called to the out-patients' departments at University College and elsewhere, and regret is expressed that the great Parisian hospital of St. Louis is entirely without such a department. The use of high-frequency electric treatment, the Finsen lamp, etc., are mentioned with approval, with the explanation that in London, "on a de l'argent," and that our institutions are complete with apparatus as with personnel, and with admirable collections in their museums. The writer considers that, clinically, the London skin hospitals are no better organised than those in Paris, but that they are superior in general organisation and in facilities for scientific research. He concludes by saying that, with improvements in these respects, the hospital of St. Louis would realise a dream of perfection.

IX.—BROMPTON HOSPITAL AND THE SANATORIUM AT FRIMLEY. By DR. VANDREMER.

As the heading of this article shows, the visitors did not see more than one of the London hospitals for consumption, and they missed the interesting features to be found at Victoria Park, Mount Vernon, and elsewhere. The writer notes the considerable decrease in tuberculous disease in England, as evidenced by recent statistics, and sets himself to ascertain the cause. It is not due to legislation, for there is none bearing on the subject. It is not due to the numbers of hospitals devoted to the treatment in London, as Brompton Hospital is, he appears to think, the only one. He describes its construction in detail, and comments on the cheerfulness of the wards, calculated to improve the "moral" of the patient, and make him forget his condition. The management and financial arrangements are discussed as well as the statistics of the cases treated annually, and comments are made on the immense value of the carefully preserved records of the hospital. The treatment of tuberculous patients in some of the general hospitals is mentioned, and the sanatorium at Frimley (not yet occupied) is fully described. Still writing, apparently, under the impression that London has only one consumptive hospital, and one sanatorium, Dr. Vandremer proceeds to discuss our charitable and philanthropic institutions in general, among which (pace Sir R. Farrant) he includes Rowton Houses, and seeks in these and in the general improvement in sanitation, dating from 1866, the reasons for the decrease in consumption. He describes London as a city furrowed with wide thoroughfares, the streets bordered with houses of moderate height, set back slightly from the roadway (by our familiar areas) so as to escape a considerable portion of the dust, and refers to the large parks and squares which separate and subdivide the various districts. All these features diminish considerably the dangers of infection. Our system of half-holidays on Saturdays, and recreation then and on Sundays, our excellent meals, our moderate hours of business, are also held responsible for our superior healthiness, and (strange to say) London is held up as a model to Paris in the absence of temptations to drink. Leaving the workshop, our workmen do not pass "terraces of cafés," for only licensed houses may provide alcoholic drink. In London one must be determined to drink, whereas in Paris an effort must be made to avoid it. Dr. Vandremer concludes by expressing the hope that the advantages which our population enjoys may be acclimatised on his side of the Channel.

X.—INFECTIOUS HOSPITALS. By DR. LOUIS MARTIN, Medecin Directeur of the Pasteur Hospital.

The writer begins by describing, with approval, our system of compulsory notification, and describes the whole system of public ambulances, disinfection, etc., adopted by the Metropolitan Asylums Board, giving a list of their hospitals, with the number of patients in each for each class of infectious disease on a given day, and compares the total, 3,579, with the total number of available beds—7,600. He contrasts the position of London, with 14 hospitals for infectious cases, with Paris, which has only that at Aubervilliers, and insists that the staff must be "instructed," as in London. To improve matters, not only good intentions are necessary, but the same means must be employed as in England, including money, and plenty of it. He points out that, at the Brook Hospital, there is more than one attendant to every two patients, and that the cost per patient is about seven francs a day. The results, however, amply justify the expense; the mortality from scarlet fever having fallen in thirteen years from 7·86 to 3·1 per cent., and from diphtheria from 33·5 to 9·6 per cent. Great surprise, however, is expressed at the exclusion of measles and whooping-cough from the official list of infectious diseases, and also at the absence of all precautions against infection on the part of the staff. The care taken at our surgical operations is contrasted with this apparent carelessness, and the question is asked "Are not medical microbes to be dreaded as much as surgical ones?" Broncho-pneumonia is, he asserts, as contagious as erysipelas. The disinfection of linen, however, is stated to be far in advance of French practice. The writer concludes by praising our practical arrangements, always excepting our "too easy" dealings with whooping-cough and measles.

XI.—GENERAL IMPRESSIONS, INCLUDING THE SUBORDINATE STAFF. By SŒUR MARIE CATHERINE, Superieure des Religieuses de l'Hôpital Pasteur.

The editor, in a prefatory note, explains that Sister Marie Catherine was specially charged to study the "curious and remarkable organisation" of our nurses, and that she is in every way qualified for the task. He adds, as this is the concluding article, a final note of thanks to his own collaborators and to his English friends for the joint result which he is able to put before his readers. The Superieure begins by saying that the English hospital is in every sense a "Home" for the poor patient, *i.e.*, as the word is untranslatable, it combines his hearth, his family, his comfort, and his physical and moral well-being. She says that the English idea is not to save money, but to assure the well-being of the patient, and that every means are employed to this end, so that London hospitals are comfortable, and even luxurious, to an extent which cannot be imagined in France. The wards are described in similar terms to those of the first article; the spaces between the beds are noted, the chimneys of faïence and the double fireplaces, the rolling furniture, and the sanitary annexes, which "leave nothing to be desired." Our system is taken as illustrative of our best national characteristics, and special comment is made on the profound liberty, which opens the hospital doors as widely to the Catholic priest as to the pastor of any Protestant sect, and permits the establishment, in every important hospital, of a chapel attended by a regular chaplain. As regards the nurses, the Superieure goes back to the days of Florence Nightingale, and traces the development of our system from her initiative. She describes the degrees of our "hierarchy," as she calls it, from matron to probationer. She notes that the individuals come chiefly from the professional classes, and that it is a concession to take one from "le haut commerce." The whole system of service, examinations and pensions is described, the proportion of nurses to patients is again stated as usually one to three, and stress is laid on the immediate detailing of a special nurse to look after any patient requiring individual attention. Our nurses' homes are described in commendatory terms, and the insistence on a high moral tone, and determination to do the best for the patient, is dwelt upon as a characteristic of English matrons and their subordinates, and as an essential which is quite as important as diplomas and degrees.

